# A fluorescence recovery after photobleaching protocol to measure surface diffusion of DAGLα in primary cultured cortical mouse neurons

**DOI:** 10.1016/j.xpro.2021.101118

**Published:** 2022-01-20

**Authors:** Sehyoun Yoon, Peter Penzes

**Affiliations:** 1 Department of Neuroscience, Northwestern University, Chicago, IL 60611, USA; 2 Department of Psychiatry and Behavioral Sciences, Northwestern University, Chicago, IL 60611, USA; 3 Northwestern University, Center for Autism and Neurodevelopment, Chicago, IL 60611, USA

**Keywords:** Cell Biology, Cell culture, Cell Membrane, Cell-based Assays, Microscopy, Molecular Biology, Neuroscience, Molecular/Chemical Probes

## Abstract

This protocol describes using fluorescence recovery after photobleaching (FRAP) of a superecliptic pHluorin (SEP)-diacylglycerol lipase α (DAGLα) to measure membrane-bound DAGLα mobility in dendritic shafts of primary cultured cortical mouse neurons. This could serve as an excellent tool to analyze endocannabinoid-mediated synaptic plasticity. We have used this protocol to show that DAGLα surface dynamics play an integral role in regulating the dendritic spine. We also detail how we test the qualities of generated SEP-DAGLα in HEK293T cells by FRAP assay.

For complete details on the use and execution of this profile, please refer to Yoon et al. (2021a).

## Highlights

• Generation of SEP-DAGLα by inserting SEP into the first extracellular domain
• Expression and functional tests of SEP-DAGLα in HEK293T cells
• FRAP of SEP-DAGLα to determine DAGLα diffusion in dendritic shafts

## Graphical abstract

**Figure:**
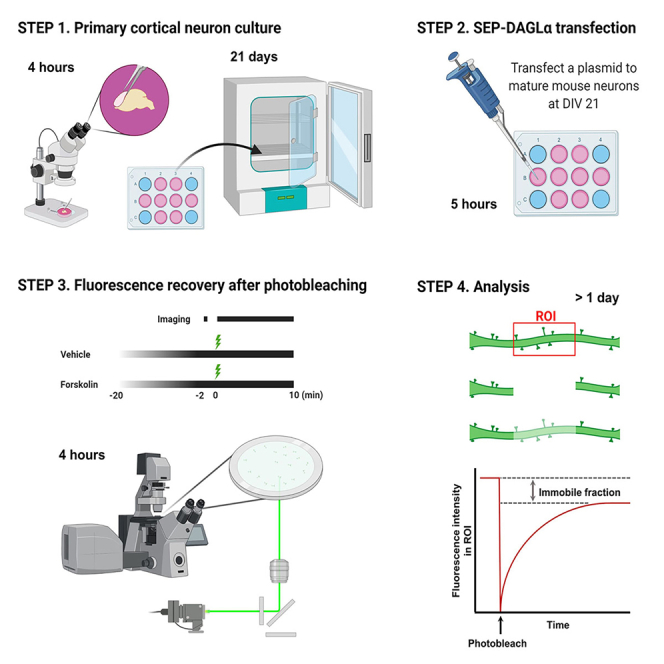

## Before you begin

**Figure 1:**
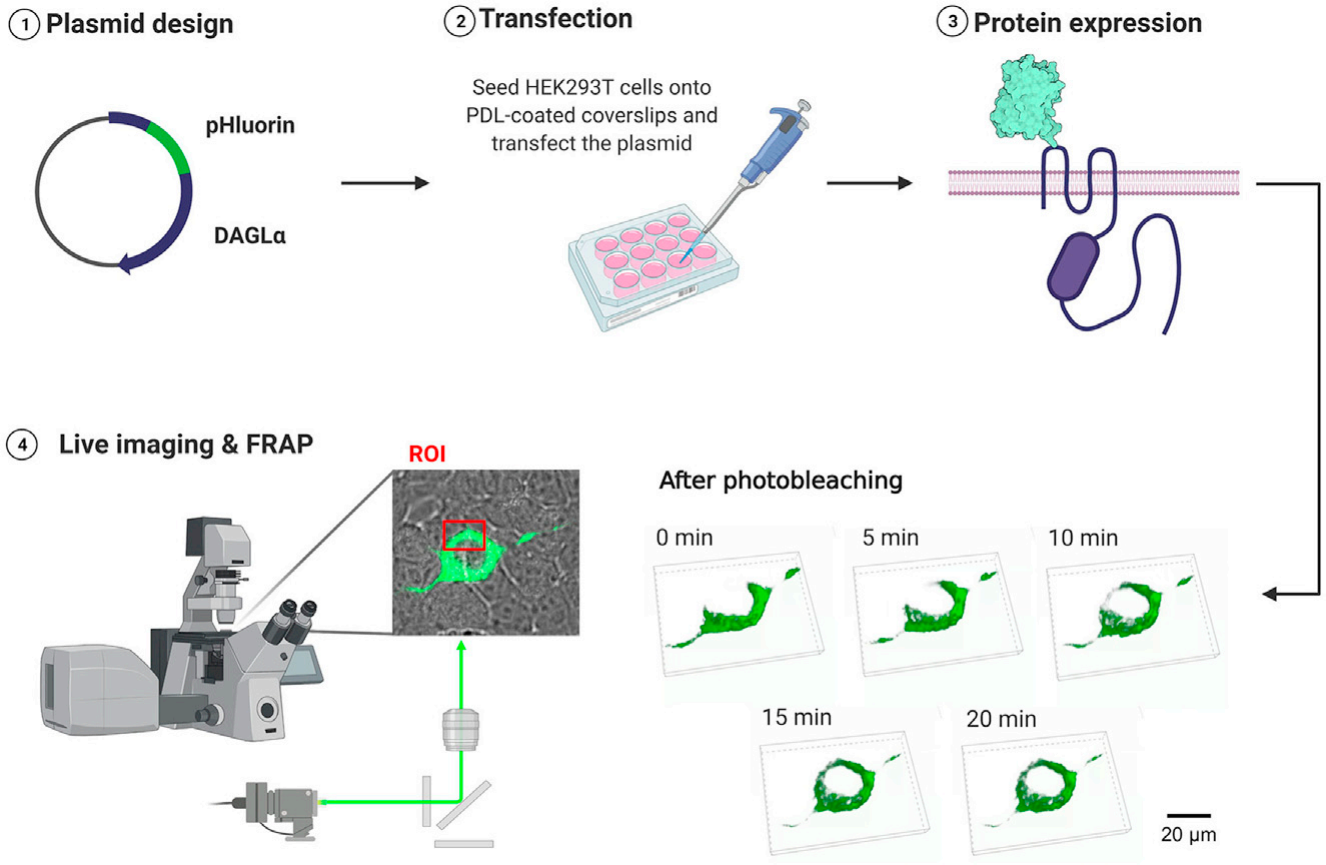
Schematic of expression and functional tests of SEP-DAGLα

The protocol describes the practical steps required for generating SEP–DAGLα by inserting SEP sequences into the first extracellular domain of DAGLα. To confirm the newly created SEP-DAGLα, the plasmid was transfected into HEK293T cells, and then the FRAP assay was performed (Figure 1). After the experiment, the sample was fixed with 4% paraformaldehyde (PFA), and the surface expression of SEP was confirmed through immunocytochemistry (Figure 5).

### Plasmid construction

**Timing: 1 week**

1. The DNA encoding full length mouse DAGLα (Entry identifier: Q6WQJ1) was fused with a superecliptic pHluorin (SEP) tag amplified from pCMV2-SEP-GluA1 (Addgene; #64942) (Yoon et al., 2021a). The SEP construct was subcloned into the first extracellular loop (amino acids 44–60) of DAGLα immediately following residue 52.

### Plasmid transfection into HEK293T cells

**Timing: 4 days**

2. Prepare poly-D-lysine (PDL)-coated coverslips or use PDL-pre-coated coverslips (GG-12-1.5-PDL; Neuvitro Corporation).
  a. Put 12 mm coverslips (No. 1.5) in a 100 mm petri dish, pour 100% ethanol, sterilize them via agitation for 10 min, place on a clean bench and turn on ultraviolet light overnight to evaporate residual ethanol.
  b. Prepared coverslips are placed in each well using autoclaved fine forceps in a 12-well dish, and then 1 mL of PDL solution (0.2 mg/mL in milli-Q distilled water) is applied per well. Incubate the dish at 37°C with 5% CO_2_.
  c. The next day, the PDL solution is collected in a 50 mL conical tube and stored at -20°C. The collected PDL solution can be reused up to 5 times within 3 months. The coated coverslips are washed 3 times with 1 mL of autoclaved milli-Q water, and then the remaining water is removed through aspiration and entirely dried by natural drying. When not in use, the prepared PDL-coated coverslips are sealed with parafilm, stored at -20°C, and can be used when needed within a week.

**CRITICAL:** Commercially available pre-coated PDL coverslips rarely show lot number variation. When you have an issue with cell adhesion for coated coverslips, you should check and discuss coating quality with the supplier.

3. Seed HEK293T cells onto PDL-coated coverslips. HEK293T cells are usually maintained in 100 mm culture dishes. When seeding, put 2% of the total number of cells on each coverslip when 80%–90% confluence is achieved on a 100 mm dish.

**CRITICAL:** HEK293T cells should not be used after passage number 20.

4. A day after seeding, transfect p3XFlag-SEP-DAGLα into HEK293T cells using polyethylenimine (PEI) transfection reagent (1 μg/μL). Incubate the transfected cells at 37°C in an atmosphere of 5% CO_2_ for 36–48 h.
  a. Prepare Dulbecco's Modified Eagle's Medium (DMEM) containing 50 μL of 2xPEI (2 μg/μL; total 100 μg) in a 1.5 mL microcentrifuge tube. Also, prepare 1 μg of p3XFlag-SEP-DAGLα with 50 μL of DMEM in another tube. After adding 2xPEI solution into DNA solution, mix them by vortexing or pipetting thoroughly. The prepared solution is incubated at 37°C for 20 min or more.
  b. During incubation, re-adjust the solution in 12 wells to 400 μL. (It is recommended to reduce the existing media rather than changing the media.)
  c. After more than 20 min, apply the prepared solution evenly drop by drop to the surface of each coverslip.
  d. After more than 4 h, replace with 1 mL of cell culture DMEM (5% FBS+ Penicillin/Streptomycin (P/S)).

**CRITICAL:** In the case of HEK293T cells, the transfection efficiency was 13.9 ± 0.4% under optimal conditions. Troubleshooting, problem 1.

***Note:*** When expressing the p3XFlag-SEP-DAGLα plasmid, the Flag tags were designed to localize to the intracellular region, and SEP was located on the extracellular surface. This structure makes it possible to confirm whether the protein expressed on the membrane is properly positioned through immunocytochemistry (Figure 5).

### Confocal parameter optimization and live time-lapse imaging

**Timing: 2–3 h**

This major step describes how to validate the laser power as well as photobleaching conditions to perform FRAP with a SEP-DAGLα.

**Figure 2:**
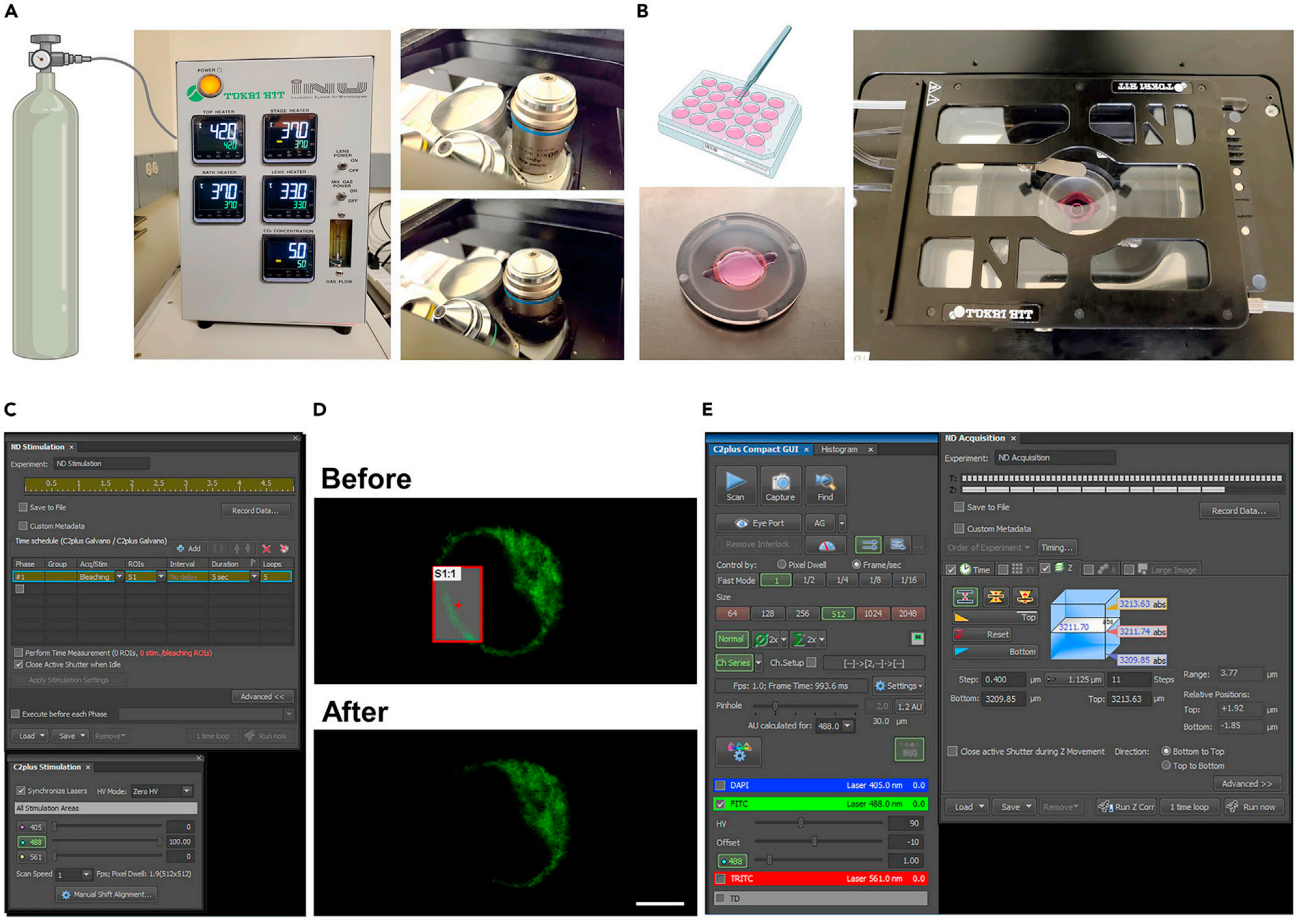
Process for FRAP imaging (A) Open CO_2_ tank valve and turn on a hit stage top incubation system. (B) Combine coverslip with a quick-release imaging chamber and place it to the live chamber. (C) Set laser intensity for photo-bleaching. (D) Representative images before or after photo-bleaching. Scale bar, 5 μm. (E) Setting for recording recovery of fluorescence intensity.

5. Two days after transfection, combine the coverslip with a quick-release imaging chamber (QR-48LP), transfer to the live imaging chamber, and prepare for imaging.
  a. Start the microscope system according to standard protocol.
  b. Turn on the Tokai Hit stage top incubation system, using manufacturer-recommended settings to maintain a temperature of 37°C in the environmental chamber, and open a 5% CO_2_ tank valve (Figure 2A).
    ***Note:*** If your incubation system is not equipped with a gas mixer, a tank of premixed gas (5% CO_2_, 20% O_2_, Balance N_2_) may be used.
  c. Check for water in a humidifier.
  d. Switch to the Plan Apo Lambda 60× 1.40 numerical aperture (NA) oil objective and attach the objective heat collar (Figure 2A).
  e. Add one drop of oil to 60× 1.40 NA objective.
  f. Switch to the Plan Apo VC 20× 0.75 NA DIC N2 dry objective.
  g. Attach environmental chamber insert to the stage base (Figure 2B).

6. Find the transfected cells at 20× magnification using a mercury-xenon lamp with a FITC filter cube.
7. Scan the entire coverslip area for healthy, brightly fluorescing cells and save each X/Y coordinate demarking a cell’s location by navigating to the ND Acquisition tab and XY sub-tab (leave XY box unchecked) and checking boxes #1, #2, etc. under Point Name.
8. Switch to the 60× 1.40 NA objective and adjust focus. If more oil is needed, carefully remove the environmental stage with the sample still securely attached to preserve X/Y positions.
9. Select a scan speed of 1 frame per second. (512 × 512 pixel resolution; pixel size: 0.14 μm)
10. Adjust the pinhole to 1.2 airy units.

**CRITICAL:** The diameter of the pinhole affects resolution in X, Y and Z. The diameter of the first minimum of the airy disk is referred to as one airy unit. The size of the airy unit at the pinhole depends on the objective lens NA, the wavelength of the fluorescent light, and any magnification up to the pinhole.

The equation for the size of the pinhole is

1 airy unit = (0.61 × wavelength × magnification)/NA

The wavelength of light is 488 nm, and the magnification is 60×. 1 airy unit is 0.128 μm. To obtain the optimal signal intensity and proper resolution from the specimen, 1.2 airy units were decided.

11. In order to bleach the SEP signal, the area of the membrane is stimulated for 5 s with 820 μW laser power (488 nm wavelength) (Figures 2C and 2D).

**Figure 3:**
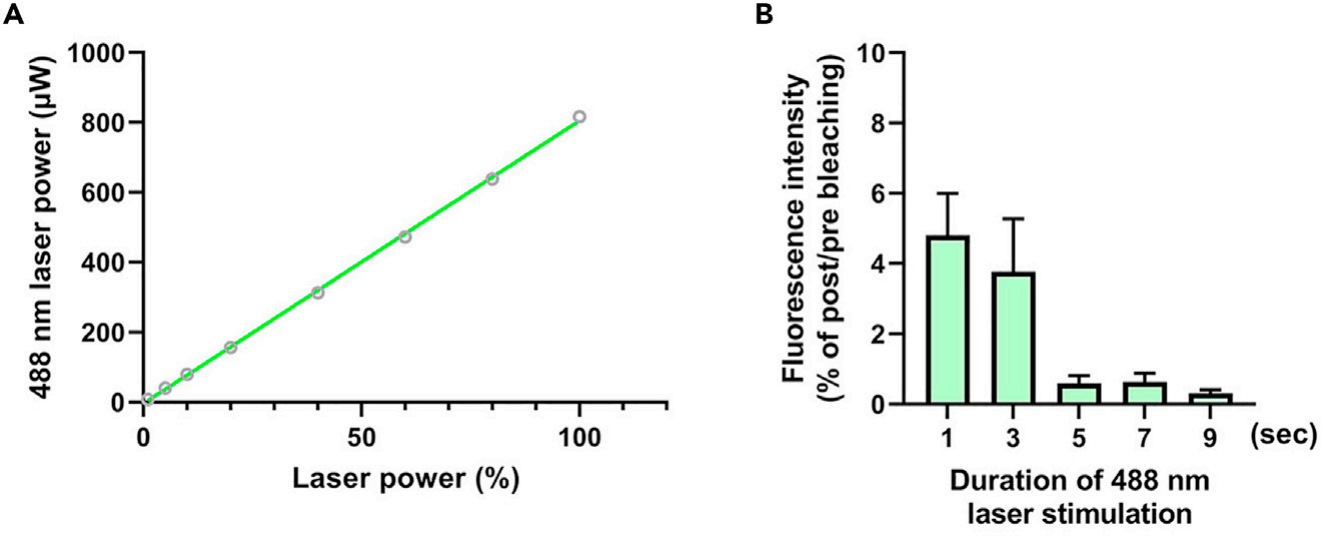
A test to determine the 488 nm laser stimulation duration (A) Confirmation of laser power (μW) according to % of 488 nm laser power in a Nikon C2+ confocal microscope. (B) The result of a photobleaching efficiency test according to stimulation duration of 488 nm laser with 820 μW intensity.

**CRITICAL:** Exposing excessive laser-light damages cells and leads to death. To obtain the maximum bleaching effect with minimal stimulation, the membrane was stimulated for different periods, and the optimal effect was confirmed at 5 seconds (Figure 3B).

**Figure 4:**
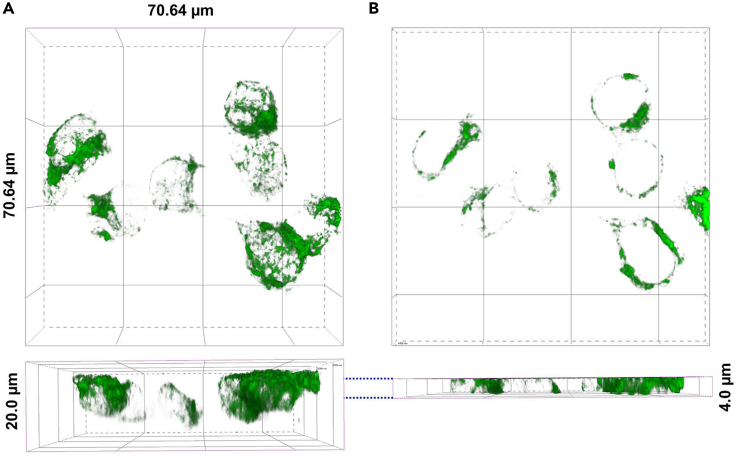
A test to determine the depth on the Z-axis (A) Representative 3D images were taken at a depth of 20 μm. (B) Representative 3D images were taken at a depth of 4 μm from (A).

12. The thickness of HEK293T cells was about 12–16 μm. Record 11 images with 0.4 μm intervals (depth: 4.0 μm) to image the membrane area (Figure 4B). At 512 × 512 pixel resolution, it takes 993.6 ms per image, each image was taken every 20 s. In addition, to investigate the appropriate recording time of the mobile fraction of SEP-DAGLα, imaging was performed for 20 min, longer than 30 s - 5 min taken in other FRAP experiments.

**CRITICAL:** Resolution depends on the diameter of the pinhole. The resolution in X and Y is determined by the distance from the center of the airy disk, and it depends on the wavelength of light (λ) and the NA. For a confocal system, the X and Y resolution equation is

Resolution of X and Y = (0.8 × λ)/(2 × NA)

and the Z resolution equation is

$$\text{Resolution of Z }=\;\left(1.4\;\times \;\lambda \;\times \;\eta \right)/{\text{NA}}^{2}$$

The wavelength of light is 488 nm, and a refractive index (η) is 1.515 for the oil lens. From the equations, the 60× 1.40 NA objective lens has 0.139 μm of XY resolution and 0.528 μm of Z resolution. To obtain optimal fluorescence intensity over the Z-plane, oversampling at 0.4 μm was performed.

13. The recorded image was converted into an AVI file to confirm that the produced SEP-DAGLα functions properly in the FRAP experiment. To create a movie file, the Movie Maker application provided by the NIS elements program was used. In a movie file produced at 3 frames per sec, the lateral diffusion of SEP-DAGLα after photobleaching was confirmed in real-time (Methods video S1).

Methods video S1. Imaging SEP-DAGLα for 20 min every 20 s after photobleaching with a 488 nm laser, related to step 13

### Immunocytochemistry and confocal imaging

**Timing: 2 days**

**Figure 5:**
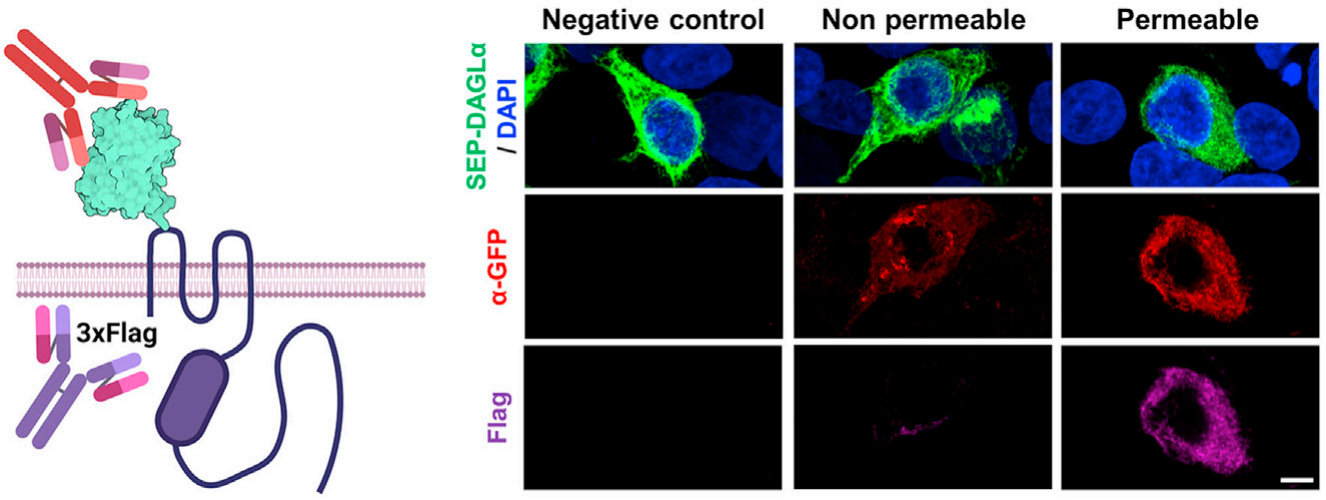
Confocal images for detecting surface-expressed SEP-DAGLα by immunocytochemistry in HEK293T cells Scale bar, 5 μm.

14. After live imaging, transfer the coverslip to a 12 well plate, and fix with 4% PFA for 10 min at 4°C with plate rocking.
15. After fixation, wash the coverslip with PBS 3 times for 5 min at room temperature (20°C–22°C).
16. After the washing step, treat with blocking solution (1% bovine serum albumin (BSA) in PBS) for an hour at room temperature on a plate rocker. Detergent is not used to detect SEP expressed on the surface only.
17. Treat each well with 500 μL of primary antibody solution (1 μg/mL) (chicken α-GFP in blocking solution), and incubate with 1° antibody overnight (16–24 h) at 4°C on a plate rocker.
18. The next day, wash with PBS 3 times for 5 min at room temperature.
19. Treat each well with 500 μL of primary antibody solution (1 μg/mL) (mouse α-Flag in the blocking solution containing 0.3% Triton X-100; 1% BSA and 0.3% Triton X-100 in PBS) and put them on a plate rocker at room temperature for an hour. Triton X-100 is employed to permeabilize the membrane, facilitating Flag-tag detection on the intracellular surface.
20. Wash the coverslip with PBS 3 times for 5 min at room temperature.
21. Add anti-mouse Alexa 647 (1 μg/mL) and anti-chicken Alexa 568 (1 μg/mL) secondary antibodies to the solution (1% BSA and 0.3% Triton X-100 in PBS) with rocking a plate at room temperature for an hour.
22. Wash the coverslip with PBS 3 times for 5 min at room temperature.
23. After being washed with PBS twice, apply DAPI (0.25 ng/mL) solution for nucleic acid staining.
24. The coverslips are finally to be mounted in ProLong Diamond Antifade Mountant onto slide glasses.
25. Obtain immunostained images with a confocal microscope (e.g., Nikon C2+ ; Figure 5). Take confocal images using the 60× oil-immersion objective (NA=1.4) as z-series of 8–10 images, at 0.4 μm intervals, with 1024 × 1024 pixel resolution (Pixel size: 0.14 μm).

## Key resources table

**Table:** 

REAGENT or RESOURCE	SOURCE	IDENTIFIER
**Chemicals, peptides, and recombinant proteins**		

DMEM; With L-Glutamine; 4.5g/L Glucose and sodium pyruvate	Fisher Scientific	Cat#MT10013CV
Fetal Bovine Serum – Heat Inactivated	Life Technologies	Cat#MT35016CV
Penicillin/Streptomycin (P/S) 100×	GIBCO	Cat#15070-063
Glutamax I Supplement 100×	Life Technologies	Cat#35050-061
D-(+)-glucose	Sigma-Aldrich	Cat#G7528-1KG
Neurobasal Medium	Thermo Fisher Scientific	Cat#21103049
B27 supplement (50×)	Life Technologies	Cat#17504044
Leibovitz's L-15 Medium	Thermo Fisher Scientific	Cat#11415064
HyClone™ Trypsin - Trypsin 0.25%	Fisher Scientific	Cat#SH3004201
Lipofectamine® 2000 Transfection Reagent	Thermo Fisher Scientific	Cat#11668019
HEPES Buffer	CORNING	Cat#25-060-CI
Trypan blue	Gibco	Cat#15250-061
Phosphate Buffered Saline (10×)	Fisher Scientific	Cat#BP3991
Poly-D-lysine	Sigma-Aldrich	Cat#P6407-5MG
Polyethylenimine	Sigma-Aldrich	Cat#408727
Ethanol	Decon Labs	Cat#2701G
Paraformaldehyde (Powder)	Millipore Sigma	Cat#158127
Triton X-100	Millipore Sigma	Cat#T8787
Bovine Serum Albumin	Fisher Scientific	Cat#BP9700100
ProLong Diamond Antifade Mountant	Thermo Fisher Scientific	Cat#P36961

**Antibodies**		

Chicken polyclonal anti-GFP (1:10,000)	Abcam	Cat#ab13970; RRID:AB_300798
Mouse monoclonal anti-Flag (clone M2) (1:1,000)	Sigma-Aldrich	Cat#F1804; RRID:AB_262044
Goat anti-Chicken 568 (1:1,000)	Thermo Fisher Scientific	Cat#A-11041; RRID:AB_2534098
Donkey anti-mouse 647 (1:1,000)	Thermo Fisher Scientific	Cat#A-31571; RRID:AB_162542

**Experimental models: Cell lines**		

HEK293T/17 cells	ATCC	Cat#CRL-11268

**Experimental models: Organisms/strains**		

C57BL6 mice	The Jackson Laboratory	RRID:IMSR_JAX:000664

**Recombinant DNA**		

pEZ-3XFlag-SEP-DAGLα	Published in Yoon et al. (2021a, 2021b)	N/A
pCMV2-SEP-GluA1	Addgene	Cat#64942
pmCherry-C1	Clontech	Cat#632524

**Software and algorithms**		

NIS Elements Version 4.30.02	Nikon Instruments	https://www.microscope.healthcare.nikon.com/products/software/nis-elements; RRID:SCR_014329
ImageJ Fiji	ImageJ Fiji	https://fiji.sc/; RRID:SCR_002285_
GraphPad Prism	GraphPad	https://www.graphpad.com/scientific-software/prism/; RRID:SCR_002798

**Other**		

Thermo Scientific SUN-SRi Luer-LOCK Syringe	Thermo Fisher Scientific	Cat#14-823-221
Syringe filter, PES, 0.22 μm	Dot Scientific Inc.	Cat#229747
Falcon® Bacteriological Petri Dishes 100 × 15 mm	Corning	Cat#351029
15 mL conical tubes	DOT Scientific Inc	Cat#416-PG
50 mL conical tubes	DOT Scientific Inc	Cat#451-PG
Filter System, 150 mL, .22μm, Pes	Corning	Cat#431153
Falcon® 40 μm Cell Strainer, Sterile	Corning	Cat#352340
Cover Glasses, Circles, 12 mm, Thickness 0.13–0.17 mm	Carolina	https://www.carolina.com/microscope-slides-covers/cover-glasses-circles-12-mm-thickness-013-017-mm/633029.pr
12-Well MultiDish Cell Culture Dish	Thermo Scientific	https://www.thermofisher.com/order/catalog/product/150200#/150200
Zeiss SteREO Discovery.V8 Stereo Microscope	Zeiss	https://www.zeiss.com/microscopy/us/products/stereo-zoom-microscopes/stereo-discovery-v8.html
Nikon C2+ confocal microscope system	Nikon	https://www.microscope.healthcare.nikon.com/products/confocal-microscopes/c2
Zyla 4.2 sCMOS	Andor	https://andor.oxinst.com/products/scmos-camera-series/zyla-4-2-scmos
CFI Plan Apochromat Lambda S 60× Oil Immersion Objective Lens	Nikon	https://www.microscope.healthcare.nikon.com/products/optics/cfi-plan-apochromat-lambda-series
CFI Plan Apochromat Lambda 20× Objective Lens	Nikon	https://www.microscope.healthcare.nikon.com/products/optics/cfi-plan-apochromat-lambda-series
Microscopy immersion oil Type F	Nikon	Cat#MXA22168
Forma II Series Water Jacketed CO_2_ incubator	Thermo Scientific	https://www.thermofisher.com/order/catalog/product/3110#/3110
Quick Release Magnetic Imaging Chamber	Warner Instruments	Cat#QR-48LP; https://www.warneronline.com/quick-release-magnetic-imaging-chambers-qr-series
Tokai Hit Environmental imaging chamber	Tokai Hit	https://www.tokaihit.com/

## Materials and equipment

Before starting primary culture, PDL-coated coverslips, high-glucose complete DMEM, L15-medium, and complete neurobasal medium (B27 supplement 1× + P/S 1× + GlutaMax 1×) should be prepared. Detailed preparation and storage methods are described in our previous paper published in STAR Protocols (Zaccard et al., 2021). All experiments were performed in accordance with the Institutional Animal Care and Use Committee at Northwestern University.

***Alternatives:*** As we mentioned above, PDL-pre-coated coverslips (GG-12-1.5-PDL; Neuvitro Corporation) could be used if the quality is tested. To perform the FRAP, similar or advanced models of Nikon C2+ confocal microscope system such as Nikon A1R, LSM 980 (ZEISS), and STELLARIS 8 (Leica) can be used alternatively. However, it is recommended to test FRAP conditions when using other systems.

## Step-by-step method details

### Primary mouse cortical neuron culture

**Timing: 4** h

1. In a biosafety cabinet, decapitate postnatal day 0 (P0) mouse pups with surgical scissors and place the head into a 100 mm petri dish filled with ice-cold L-15 medium.
2. Remove the scalp and immerse the brain in a new petri dish containing cold L-15 medium using forceps. The plate containing the brain is placed on ice and stored until cortical neurons are obtained.
3. Using a stereomicroscope, remove the meninges with forceps, remove the cortices, and transfer them to a 15 mL conical tube. Usually, up to 2 brains are placed in one 15 mL conical tube and kept cold on ice.
4. Sterilize the 15 mL conical tube containing the cortices with 70% ethanol and transfer to the biosafety cabinet. Carefully remove the L-15 medium using a pipette, add 300–400 μL of pre-warmed 0.25% trypsin, and incubate at 37°C for 10 min.

**CRITICAL:** Never exceed 12 min for 0.25% trypsin incubation at 37°C. Extended incubations decrease the survival rate of neurons.

5. After incubation, re-sterilize the conical tube with 70% ethanol and put back into the biosafety cabinet. Tilt the conical tube and carefully remove trypsin with a pipette, and fill with 1 mL of pre-warmed high-glucose complete DMEM.
6. Mechanically dissociate cortical tissue to individual cells using a pipette.

**CRITICAL:** Trypsin treatment for 10 min digests various extracellular matrix proteins allowing easy mechanical dissociation. However, neurons are weakened and can die quickly. To increase the survival rate of neurons, resuspend cells by pipetting up and down with a 1 mL tip placed close to the bottom of the conical tube. Pipetting should be done gently and carefully, never performing more than 10 repetitions.

7. After adding 4 mL of high-glucose complete DMEM, filter it using a 40 μm nylon cell strainer into a 50 mL conical tube.
8. Transfer 10 μL of solution from a 50 mL conical tube to an Eppendorf tube and add 10 ul of trypan blue solution. Count live cells using a hemocytometer and calculate the number of cells per mL in a 50 mL conical tube.
9. Seed 1.333 × 10^5^ cells/cm^2^ on the PDL-coated coverslip. Fill each well of a 12-well plate with up to 1 mL of the high-glucose medium.

**CRITICAL:** The 4 wells in the corners of the 12 well plate are filled with autoclaved distilled water to mitigate evaporation of the medium. Troubleshooting, problem 2.

10. Transfer the 12-well plate to the incubator at 37°C with 5% CO_2_ and wait for 2 h for neuronal precursor cells to settle on the PDL-coated coverslip.
11. After 2 h, the adhesion of the precursors to the coverslip is checked using an epi-illumination microscope. Carefully remove high-glucose complete DMEM at the biosafety cabinet and replace with 1 mL of pre-warmed complete neurobasal medium. Troubleshooting, problem 3.
12. Maintain the prepared cortical neuronal culture in the incubator at 37°C with 5% CO_2_ for 21 days.

### Maintenance of primary cortical neuron culture

**Timing: 15–30 min, every 3 or 4 days until DIV 21**

13. Replace the complete neurobasal medium twice a week.

### SEP-DAGLα transfection into primary cultured cortical neurons

**Timing: 5 h**

14. Prepare the solutions for transfection in advance.
  a. Transfection neurobasal medium (B27 supplement 1× + GlutaMax 1×)
  b. H-DMEM (HEPES pH7.2–7.6 added to a final concentration 10 mM in DMEM)
  c. Lipofectamine 2000 transfection reagent
  d. pEZ-3xFlag-SEP-DAGLα plasmid and pmCherry-C1 plasmid
  e. 12-well plates filled with 400 μL of transfection neurobasal medium and placed into the incubator at 37°C with 5% CO_2._

**CRITICAL:** Transfection media must be free of antibiotics to prevent neuronal cell death.

15. Prepare two different Eppendorf tubes in the biosafety cabinet. Prepare 50 μL per sample of h-DMEM in 1 tube and transfection neurobasal medium in the other tube. Mix 2 μg total of plasmids (ex. 1 μg of SEP-DAGLα + 1 μg of mCherry) in transfection neurobasal medium, and 2 μL per sample of lipofectamine 2000 in the other h-DMEM.
16. After mixing the h-DMEM solution with transfection neurobasal medium through pipetting, incubate for 20–30 min at 37°C.
17. While waiting for 20 min, transfer the coverslips to a 12-well plate filled with 400 μL of pre-warmed transfection neurobasal medium using autoclaved fine forceps.

**CRITICAL:** The 12-well plate and medium used for maintenance of the neuronal cells put back into the incubator. After transfection, the coverslips will be returned to the original 12-well plate.

18. After incubation for 20–30 min, treat 100 μL of transfection solution on each coverslip drop by drop using a 200 μL tip and place in an incubator for 4 h.
19. After 4 h, return the coverslip to the original 12-well plate using autoclaved fine forceps and place it in the incubator.
20. Incubate dishes in a humidified incubator at 37°C with 5% CO_2_ for ∼48 h to allow time for adequate fluorescent protein expression.

**CRITICAL:** In the case of mouse cortical primary cultured neurons, the transfection efficiency was 0.03% under optimal conditions.

### Live time-lapse imaging with a confocal microscope

**Timing: 4 h**

21. Two days after transfection, combine the coverslip with a quick-release imaging chamber (QR-48LP) and securely mount the chamber onto the environmental chamber stage as described above.
22. The entire area is searched by 20× magnification, and the positions of the transfected pyramidal neurons are saved through X/Y coordinate demarcation.

**Figure 6:**
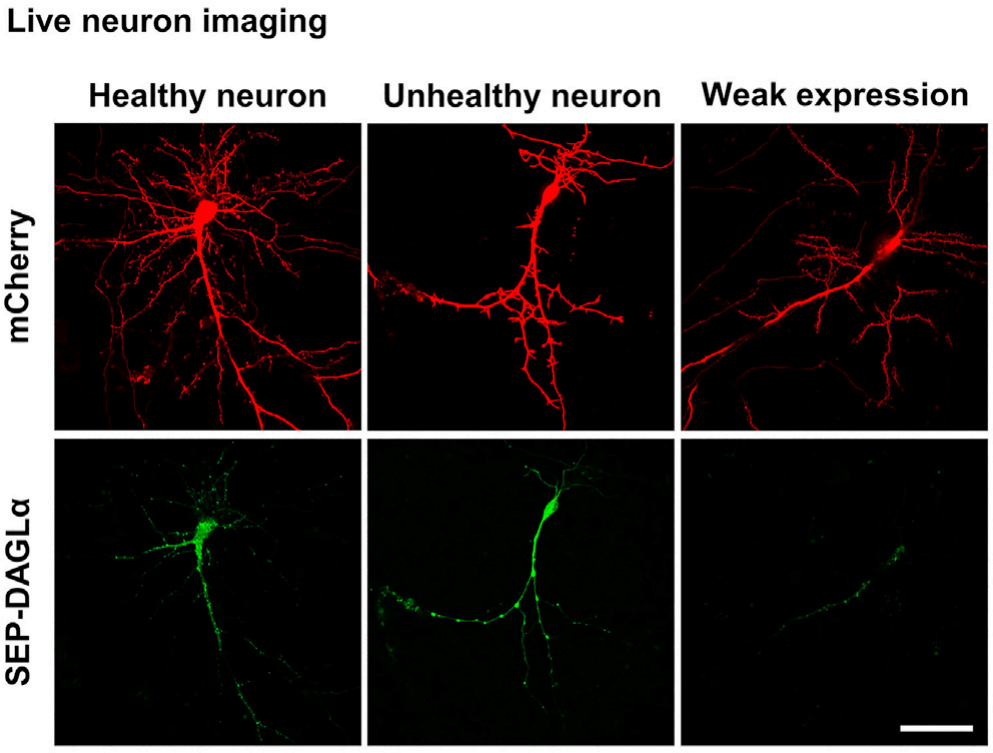
Representative live images of a healthy neuron (left) and an unhealthy neuron (center) A healthy neuron that weakly expresses SEP-DAGLα (right) is also shown. Scale bar, 50 μm.

**CRITICAL:** To ensure experimental reproducibility, only healthy pyramidal neurons are considered, and subjects with abnormal morphology or weak fluorescence intensity are excluded (Figure 6). Troubleshooting, problem 4.

23. Switch to the 60× 1.40 NA objective lens and adjust focus. Navigate designated neurons by the ND Acquisition tab and XY sub-tab (Figure 7A).
  a. Adjust the pinhole to 1.2 airy units.
  b. Select a pixel size as X = 0.17 μm, Y = 0.17 μm for the 60× 1.40 NA lens, and a scan area of 256 pixels × 256 pixels.
  c. To identify the strongest signal Z-plane that can be detected in the dendrite, 7 images over a 1.5 μm section were taken at 0.25 μm intervals. 4 images corresponding to the 1.0 μm section were chosen based on fluorescence intensity for measurement (Figure 7A).
  d. Adjust the laser power to 8 μW, photomultiplier tube (PMT) gain (110 for 488 nm laser and 120 for 561 nm laser; named as HV by the software), and offset (0) for each channel to achieve optimal signal-to-noise (Figure 7B).
  e. Use scan mode to adjust the field of view, centering on the secondary branches of apical neuronal dendrites.

**Figure 7:**
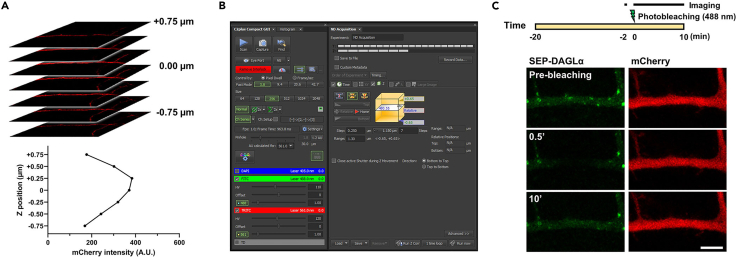
A step-by-step flowchart for FRAP analysis (A) mCherry intensity for each layer in a dendritic shaft. (B) Set the confocal microscope for imaging and FRAP. (C) Schedule and region of interest for FRAP. Scale bar, 5 μm.
24. Take images of mCherry and SEP-DAGLα in dendrites with 2 channels (488 and 561 nm) at the same time before bleaching. mCherry intensity is used for the normalization of SEP-DAGLα intensity.
25. Two min after the image is taken, a 488 nm wavelength laser is used to bleach the SEP signal in the dendritic shaft, centered on a single z-section with the strongest SEP-DAGLα intensity (Figure 7C).
  a. With ND stimulation menu, set the region of interest (12 μm (width) × 8 μm (height)). Troubleshooting, problem 5.
  b. To bleach the SEP signal, the center of the dendrite is stimulated for 5 s with 820 μW laser power (488 nm wavelength). This condition was confirmed by a previous experiment with HEK293T cells.
26. Immediately after photobleaching, record 7 images-Z-stacks with 2 channels (488 and 561 nm) at 0.25 μm intervals, every 30 s, 21 times for 10 min. Based on HEK293T FRAP recovery times, a 10 min imaging period was performed.
27. Analyze FRAP by GraphPad Prism 9 from the recorded information. Detail methods are described in Quantification and statistical analysis.

## Expected outcomes

**Figure 8:**
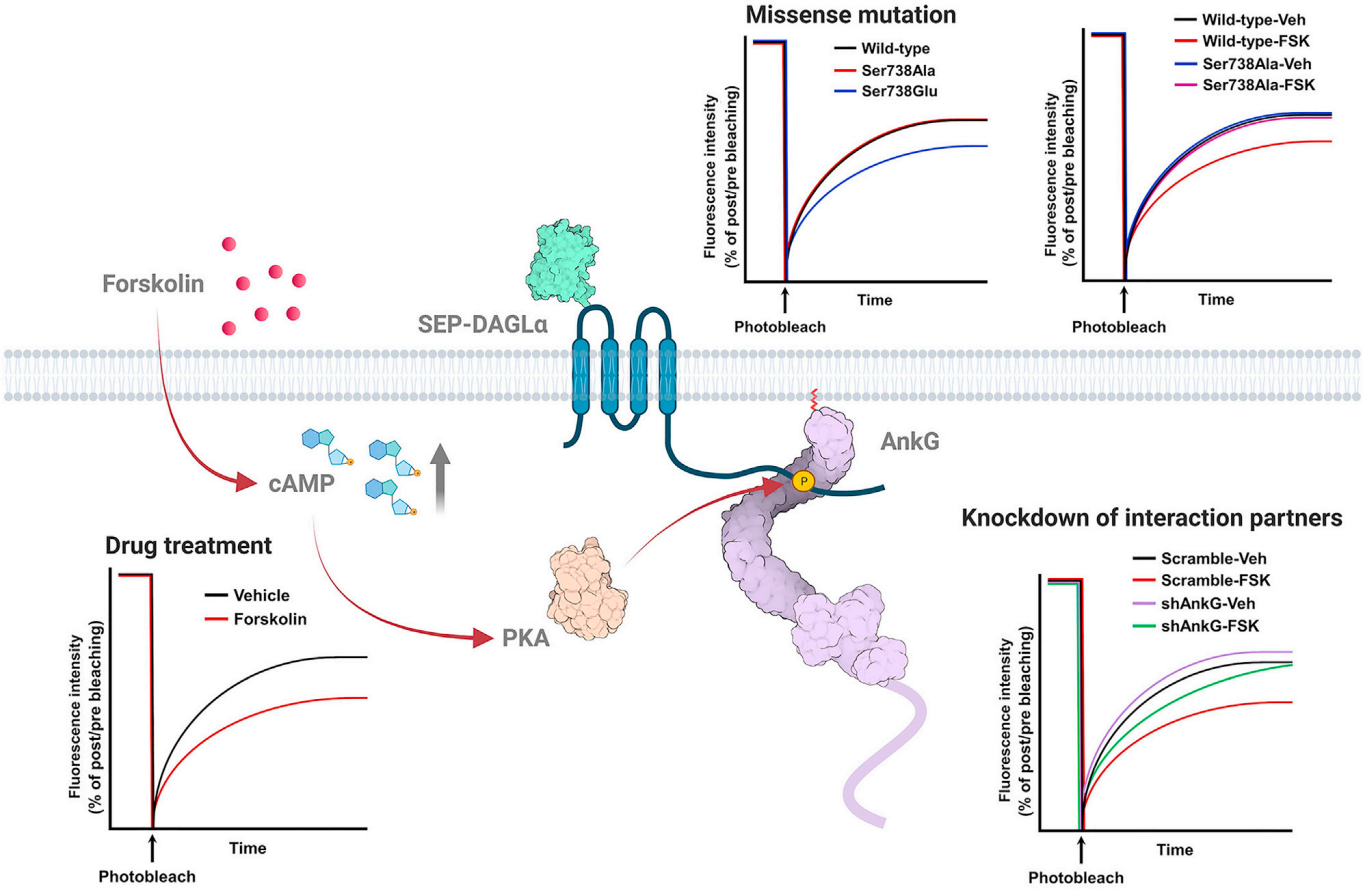
Experimental examples that can be analyzed through FRAP using SEP-DAGLα Modified by permission from Elsevier (Yoon et al., 2021a).

If the experiment is performed successfully, this technique will allow researchers to observe the diffusivity of DAGLα in the dendritic shaft. Note, precise photobleaching of dendritic spines can also allow FRAP imaging of synaptic DAGLα. A previous study showed forskolin treatment increased intracellular cyclic adenosine monophosphate (cAMP) levels, activating protein kinase A (PKA) signaling, and triggered a signaling cascade leading to DAGLα serine 738 phosphorylation (Yoon et al., 2021a). Forskolin treatment-induced serine 738 phosphorylation of DAGLα enhances its interaction with ankyrin-G, increasing spine size and decreasing DAGLα surface diffusion. Using this tool, researchers can assess the role of DAGLα dendritic membrane mobility on synaptic alterations (Figure 8).

A recent report has determined three heterozygous rare variants in human *DAGLA* (His810Gln, Arg815His, Ala858Val) close to ankyrin-G interaction sites, and the mutations were significantly related to neurodevelopmental disorders such as seizures, autism, and abnormalities of brain morphology (Smith et al., 2017). Also, alterations in ankyrin-G levels, induced by miR-34a dysregulation (Bavamian et al., 2015) or single nucleotide polymorphism (Hughes et al., 2016, 2018), are significantly linked to bipolar disorder and schizophrenia. FRAP assessment using SEP-DAGLα is expected to be an essential tool for interpreting the etiology associated with psychiatric disorders.

The EVH1 domain of Homer1b/c, highly enriched in PSD, is responsible for the regulation of spine morphology and the maintenance of a stable ankyrin-G localization in spine heads (Yoon et al., 2021b). DAGLα is known to interact with Homer1 through its C-terminal region. This FRAP assay may therefore lead to a clear understanding of the synaptic localization and dynamics of DAGLα, and the impact of this on Homer1b/c complex formation within spine heads.

## Quantification and statistical analysis

**Timing: 3–6 days**

1. Open FRAP images using ImageJ software.
2. Draw a region of interest based on the bleached area. Press Ctrl + t or open the ROI Manager (Analyze → Tools → ROI Manager), and press the Add [t] button to record the specified area.
3. Copy the region and place it in a background area. Later, the value measured in the background is used to correct the default value of fluorescence intensity.
4. Designate fluorescence intensity measurement using Set Measurements (Analyze → Set Measurements → select Mean intensity).
5. Click Multi Measure and choose the strongest intensity plane at each time point. Repeat this step to measure the fluorescence intensity of a background in non-fluorescent region (Figures 9A and 9C).

**Figure 9:**
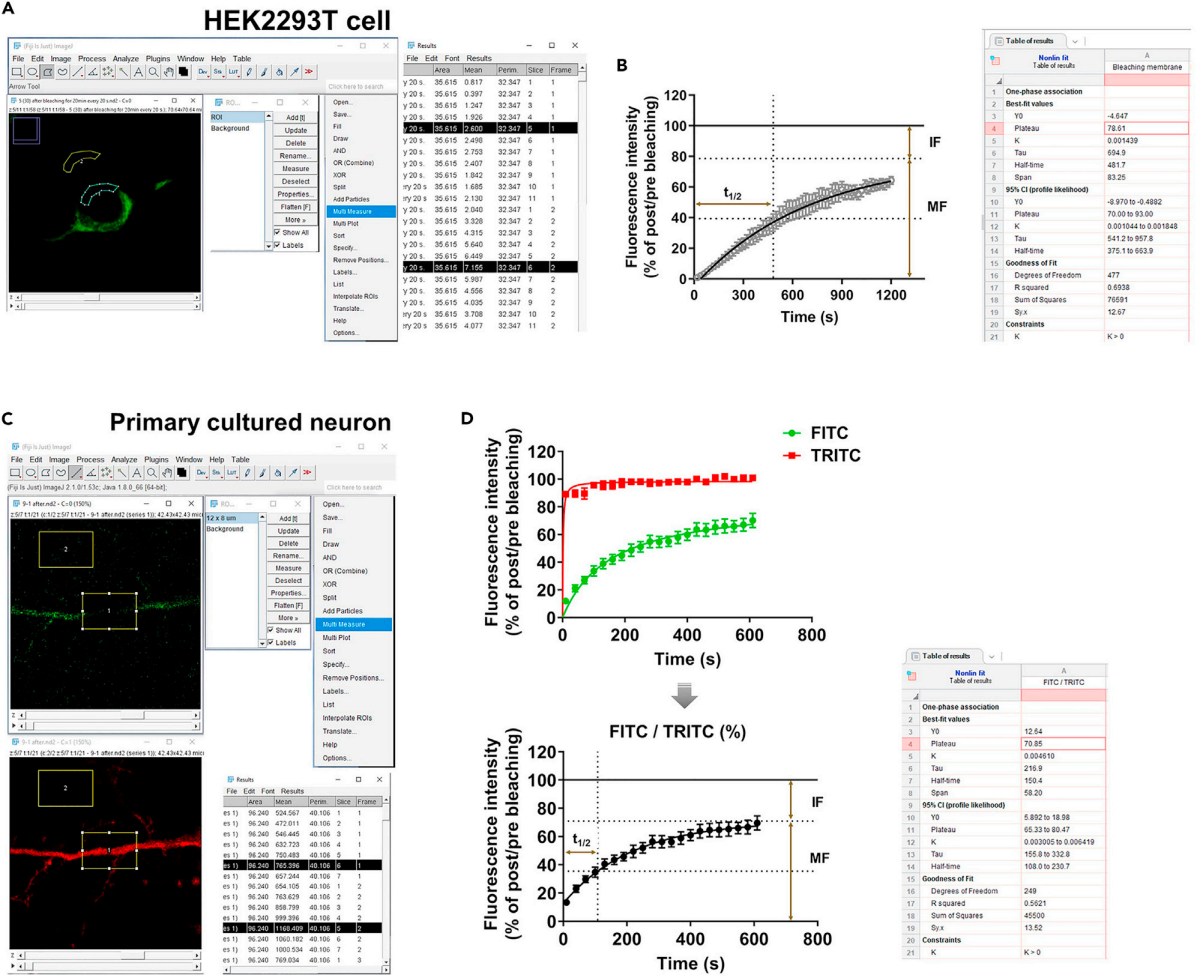
Process for analyzing fluorescence intensity (A) Individual settings to analyze fluorescence intensity in the region of interest of a HEK293T cell using ImageJ. (B) FRAP curve of SEP-DAGLα in HEK293T cells. (C) Individual settings to analyze fluorescence intensity in the region of interest of a primary cultured neuron using ImageJ. (D) FRAP curve of SEP-DAGLα in primary cultured neurons. Normalize the intensity of FITC by TRITC. One-phase exponential equations fitted the curves. t_1/2_: the half time of equilibrium; IF: immobile fraction; MF: mobile fraction.
6. Normalize the fluorescence intensity at each time point by subtracting the background values to remove experimental noise, and divide all values by the pre-bleached baseline measure. The obtained results are converted to % max intensity, based on the pre-bleached baseline.
7. Open GraphPad Prism. Select New table & graph → Grouped table. Import the results (X-axis: time; Y-axis: fluorescence intensity).
8. To analyze curve fit, click the Analyze and choose XY analyses → Nonlinear regression (curve fit) → Exponential → One-phase association.
9. From the table of results, it is possible to check the immobile fraction vs mobile fraction proportion from the Plateau values. It is also possible to calculate the half-life (t_1/2)_ value, which is the time it takes to reach half-maximal fluorescence intensity from the plateau value (Figures 9B and 9D).
10. The advantage of this analysis is that it can measure changes in the mobile fraction and half-life of equilibrium under varying conditions, such as drug treatment, SEP-DAGLα mutations, or knockdown of an interaction partner (Figure 8).

## Limitations

The main limitation of FRAP measurements is the low image resolution possible within the imaging period, given that multiple Z-planes and channels must be imaged within the short recovery time. As the bleached volume is usually determined by the radial and axial resolution of the microscope, the recovery rate is confirmed by the measurement of the strongest optical intensity by observing the three-dimensional change.

Next, photodamage can be potentially problematic in FRAP experiments, due to the high laser power needed for pre-photobleaching. Therefore, we illuminate the laser under various conditions to reduce potential photodamage. To ensure the health of the cells being imaged, we test for potential photodamage and check for morphological changes in the dendrites of neurons. To achieve this, brightfield and mCherry fluorescence images of cells are taken together before and after 488 nm laser illumination. These images are then compared to evaluate the cells for dendrite thickness, spine morphology, generation of large vacuoles, or aggregation of fluorescent proteins.

## Troubleshooting

### Problem 1

Low transfection efficiency in HEK293T cells (step 4)

### Potential solution

To achieve the best transfection efficiency, it is recommended to transfect at ∼60% confluence of HEK293T cells. HEK293T cells should be seeded the day before at 30% confluence. Seeding cells at 15%, two days before is not recommended because it reduces transfection efficiency.

### Problem 2

Contamination of samples during primary mouse cortical neuron culture (step 9)

### Potential solution

Primary culture is the process of transferring neurons from in vivo to in vitro, and exposure to numerous contaminated environments can occur. Thus, maintenance of a sterile environment is crucial to ensure cell health over the 3 week maturation period.

P/S is added to all growth mediums (except for transfection medium). It is also recommended to add antimycotic agents to protect from fungi and yeasts. However, since high concentrations are toxic, particularly to neuronal cultures, conducting a toxicity test based on manufacturer's guidelines is recommended. High-glucose complete DMEM and complete neurobasal medium are vacuum-filter sterilized through a 0.22 μm pore size. Latex gloves, dedicated neuronal culture lab coat and mask should be worn during the experiment. Sanitize stereomicroscope, all items, and gloved hands in and out of the biosafety cabinet with 70% ethanol.

### Problem 3

Decrease in survivability or health of neurons after primary culture (step 11)

### Potential solution

As described above, various factors increase neuronal cell death in vitro.

The researcher should use mouse pups immediately after birth. To ensure the breeding of P0 pups, we recommend mating the male and female only for 1 night and separating them the next morning. Check the mouse cage every morning after 19 days. Most C57BL6 mice tend to be born between the 19th and 21st days after fertilization.

Next, we recommended that the entire process from decapitating the pup’s heads to obtaining cortical tissue under a stereomicroscope (just before trypsin treatment) should be done within an hour. Experienced researchers may get cortical tissues from one pup every 7 min. So, it is recommended to perform primary culture with about 8 pups in one experiment at maximum. Sample collection exceeding 1 h and 20 min adversely affects the survival rate of neuronal cells. Trypsin treatment time should also not exceed 10 min.

Lastly, neuronal cells are weakened after trypsin treatment, so proceed with physical dissociation slowly, by pipetting 10 times maximum.

### Problem 4

Determination of time to achieve proper fluorescence intensity of mobile fraction (step 22)

### Potential solution

The timing to achieve maximum fluorescence intensity after photobleaching varies due to differences in cell types, sample thickness, and transfection efficiency. Thus, it is necessary to check the optimal duration of imaging according to the experimental conditions. Based on initial trials imaging of 2 neurons over 20 min, the optimal duration was determined to be 10 min.

### Problem 5

Determination of surface diffusion of DAGLα in primary cortical neurons (step 25)

### Potential solution

After photobleaching, fluorescence is restored by lateral diffusion trafficking to the surface and exocytosis translocating DAGLα from the cytoplasm to the membrane. To analyze the fluorescence recovery rate by exocytosis the entire HEK293T cell body expression SEP-DAGLα was bleached with a 488 nm laser (Yoon et al., 2021a). It was confirmed that fluorescence intensities of 0.77% at 10 min and 1.11% at 20 min were recovered. Based on these results, the effect of recovery by exocytosis in the experiment to confirm lateral diffusion is insignificant. Moreover, the effect of exocytosis can be effectively excluded by narrowing the bleaching area.

## Resource availability

### Lead contact

Further information and requests for resources and reagents should be directed to and will be fulfilled by the lead contact, Peter Penzes (p-penzes@northwestern.edu).

### Materials availability

Further information and requests for materials should be directed to and will be fulfilled by the lead contact, Peter Penzes, upon request. All unique/stable reagents generated in this study are available from the lead contact with a completed Materials Transfer Agreement.

## Data Availability

This study did not generate any custom code, software, or algorithms. Datasets related to the current study are available from the lead contact, Peter Penzes, upon request.
